# Assessment of the Highest Stress Concentration Area Generated on the Mandibular Structure Using Meshless Finite Elements Analysis

**DOI:** 10.3390/bioengineering7040142

**Published:** 2020-11-08

**Authors:** Andrea Fabra Rivera, Frederico de Castro Magalhães, Amalia Moreno, Juan Campos Rubio

**Affiliations:** 1 Department of Mechanical Engineering, Engineering School, Universidade Federal de Minas Gerais, CEP 31270-901 Belo Horizonte-MG, Brazil; andreadelpilar65@gmail.com (A.F.R.); fredmag.castro@gmail.com (F.d.C.M.); 2 Department of Oral Surgery, Pathology and Clinical Dentistry, School of Dentistry, Universidade Federal de Minas Gerais, CEP 31270-901 Belo Horizonte-MG, Brazil; Amalia_moreno@yahoo.com.br

**Keywords:** finite elements methods, meshless software, dental prosthesis fixing, geometric complexity problems

## Abstract

Frequently, the oral cavity area can be affected by different diseases, so the patient needs to be submitted to surgery to remove a specific region of the mandibular. A complete or partial discontinuity of the mandibular bone can cause direct or indirect forces variations during the mastication. The dental prosthesis is an alternative to generate an aesthetic or functional solution for oral cavity lesions. However, they can be wrongly designed, or they can lose the adjustment during their useful life, deteriorating the patient’s condition. In this work, the influence of the fixation components position for a dental prosthesis will be studied based on the finite element method. By means, it is possible to determine the area of the highest stress concentration generated on the mandibular structure. The temporomandibular image obtained by computational tomography was used as a 3D graphic whole model because in the medical area the morphological factors are extremely important. Vertical loads of 50, 100, 150 and 200 N were applied in three different regions: in the whole buccal cavity, simultaneously in the left and right laterals and only in the right lateral, to determine the values of von Mises stress in the mandible. These results were compared between three finite element software packages (Ansys^®^, SolidWorks^®^ and Inventor^®^) and a meshless software (SimSolid^®^). They showed similar behaviors in the highest mechanical stress concentration in the same regions. Regarding the stress values, the percentage error between each software package was less than 10%. The use of SimSolid^®^ software (meshless) proved to be better at identifying the higher stress generated by the dental prosthesis in the facial skeleton, so its computational efficiency, due to its geometric complexity, was highlighted.

## 1. Introduction

Several clinical factors make the removal of a specific mandibular area affected by osseo-mandibular diseases necessary [1]. The presence of carcinogenic tumors, infectious diseases, congenital anomalies and traumatic injuries in the oral cavity area usually causes the complete or partial remotion of the mandibular bone [2].

Another factor to be considered is that the mandibular plates, dental prosthesis or implants that will to be inserted and fixed into the mandibular bone will allow the choice of the most adequate place to perform the insertion and fixation [3].

The replacement of an organ or a body member by a prosthesis which represents for the patient recovery and the capacity to perform some tasks again, and it is similar to the mandibular surgery process as the surgery linked with the dental prosthesis fixation aims to restore the mastication, phonation, swallowing and aesthetic of the patient [4].

Consequently, when a mandibular surgery is performed, it also changes the patient’s anatomy by losing totally or partially the teeth in the procedure when there is alveolar ridge resorption after its extraction [5].

When this reabsorption occurs, the mastication functions are affected. Normally, it is characterized by a decrease in the mastication force and a change of the bite region. Further, the use of a conventional removable prosthesis does not allow adequate healing of the masticatory function of the fully edentulous mandible. Barone et al. [6] affirmed that to guarantee the restoration of essential functions, both mastication and phonation, of the patient, a fixed prosthesis aided by dental implants must be used.

In terms of the jaw anatomy, the choices of the place to fix the plates and dental prostheses can be severely affected by creating an asymmetric fixation [7]. As mentioned by Schuller-Götzburg et al. [8], the patient oral cavity size and geometry, the mucosa thickness and the mandibular bone and teeth condition must be identified. Maspero et al. [9] conducted a study where cone-beam computed tomography (CBCT) and bi-dimensional reconstructed lateral cephalograms (RLCs) were implemented to evaluate the length and growth of the mandibular body. The surgery success depends on the anatomical details, direction and extent of the necessary displacement, and the experience of the surgical team [10]. Farronate et al. [10] developed an experimental protocol to optimize presurgical orthodontic planning using the method of linear and rotational discrepancies of skeletal structures.

Likewise, the importance of evaluating the mechanical behavior generated by a dental prosthesis, according to Cervino et al. [11], is that it allows the facial skeleton mechanical stress concentration to be estimated, so it reduces the injuries caused by unnecessary efforts or overloads. Among the numerical techniques, the finite element method (FEM) was promising. This numerical technique bases on the discretization of a geometric domain of interest using finite elements. According to Singiresu et al. [12], the resolutions of differential equations generate an approximate solution for a physical system, which can be minimized by an error function.

In dentistry, finite element analysis (FEA) can be applied in different subject matters, especially when focused on the development of complex biomechanical problem solutions, such as implantology, orthodontics, maxillary orthopedics, dental prosthetics and maxillofacial surgery, among others [13].

Cicciù et al. [14] carried out a study on the distribution of stress in the mandibular bone due to an overdenture fixed by dental implants, conducted with the finite element method. The researchers used a human edentulous skull model obtained by computed tomography and created a CAD jaw virtual model. The dental implants were evaluated on how their location can influence the masticatory stress distribution, both on the bone tissue and the screwed mandibular prosthesis recovery. In these numerical studies, a high computational cost was observed for the discretization of geometry, which, depending on its complexity, can make the study unfeasible or even generate inaccurate results. [15].

Finally, this study was carried out to show the reliability of the use of SimSolid^®^ software in the dentistry area, due to the complex geometries that were obtained, allowing the production of biomechanical studies that are of great interest and the study of how different computational tools can be implemented in this area.

## 2. Methods and Materials

According to the severity of the clinical case exposed and the region compromised by a disease, the jaw area must be removed, so the patients can be classified as fully or partially edentulous. In this study, there were three (3) clinic case simulations which had congenital anomalies and traumatic injuries in the lower jaw arch.

**Figure 1:**
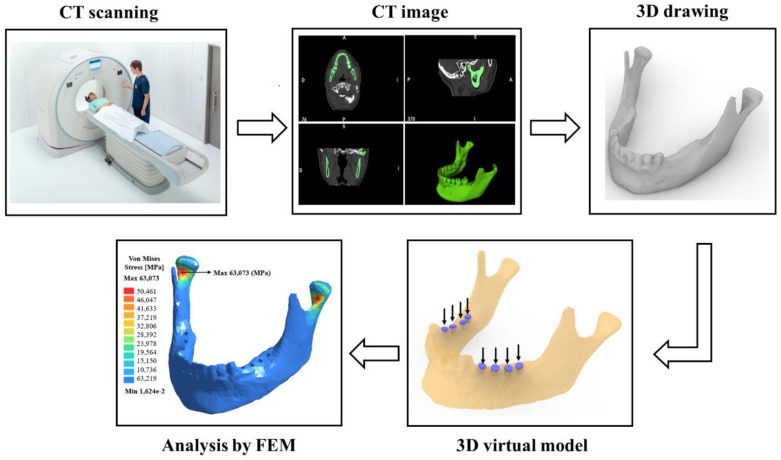
The sequence of the steps involved to use 3D maxillofacial images from the computed tomography scan, and the application of the finite element method (FEM)-based stress analysis.

Figure 1 shows a brief schematic diagram, used to create a methodology for a digital mandible reconstruction from a computer tomography. After that, the file obtained was converted to a stereolithography (STL) file or a 3D draw format, and later it was possible to perform the stress analysis by the finite element method.

(a) Computed tomography (CT) scanner [16];
(b) Image obtained by CT of the mandible in the axial, sagittal and coronal plane and a 3D view;
(c) A 3D digital model (drawing) of the mandible;
(d) A virtual planning of the implants distribution;
(e) The comparison between finite element software packages for stress analysis to be used in the dental area.

Figure 1 shows an example of the adopted methodology applied for a partially edentulous patient on both the right and left sides of the lower dental arch.

**Figure 2:**
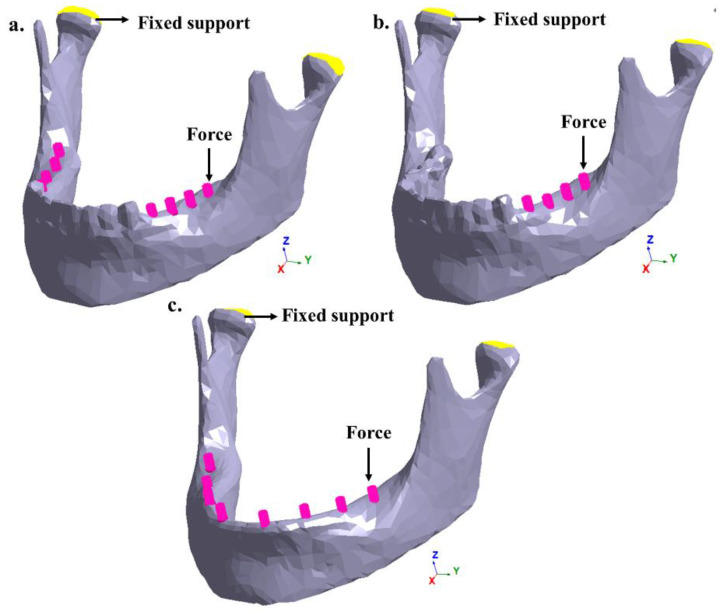
Boundary conditions and chewing forces: (**a**) the right and left lateral areas of the lower dental arch, (**b**) the right lateral area of the lower dental arch and (**c**) the total area of the lower dental arch.

Figure 2a shows a partially edentulous patient on the right and left sides of the lower dental arch. Figure 2b also shows a partial edentulism, where the right lateral area of the dental arch is affected. Finally, Figure 2c shows a totally edentulous patient. In the three studied cases, the insertion of retention pins (Muchor^®^) was simulated based on the available area and location of each one in the mandibular bone [17].

Case studies:

(a) Patient with congenital anomalies areas in the right and left sides of the lower dental arch;
(b) Patient with congenital anomalies in the right lateral area of the lower dental arch;
(c) Patient with total congenital anomalies in the lower dental arch.

### 2.1. Materials Properties

The cortical bone and teeth were assumed to be linear, isotropic, elastic and homogeneous materials. This simplification was considered to be sufficient for this study. The materials properties used in the simulations are listed in Table 1.

### 2.2. FE Mesh

The model geometry and dimensions were based on the general morphology of natural humans. The 3D models of the whole mandible previously observed were analyzed using the FEM and the new approach, meshless. Part of the cortical bone and the teeth that make the dental arch of the mandible can be seen in this study.

The finite element computational codes use different mesh generation algorithms, sizes and types of elements according to the geometry and dimension of the physical problem. Thus, the use of an adequate mesh is the main factor for an accurate numerical simulation [20]. For this reason, meshing algorithms are available to solve problems with specific requirements such as boundary conditions, mechanical properties (anisotropic materials), geometric shapes to be analyzed and discretization solvers. For numerical simulation software packages, Ansys^®^ (17.2, Ansys, Canonsburg, Pennsylvania, USA), SolidWorks^®^ (2014, Dassault Systèmes, Waltham, MA, USA) and Inventor^®^ (2019, Autodesk, San Rafael, CA, USA) were used to generate the tetrahedral volumetric (four-node linear) mesh from the triangle surface mesh. After the mandible was discretized, a localized refinement of the mesh was necessary. The local refinement was used to reduce the computing cost and also keep a good numerical accuracy in the regions of interest as well, but it requires careful system design. Thus, for the mesh refinement in the traditional software (Ansys^®^ SolidWorks^®^ and Inventor^®^), a mesh control was made after concluding the initial simulation. The mesh refinement was established mainly by the size decrease of the element and, consequently, an increase in the total number of nodes and elements in the areas where the highest stress concentration was observed [21]. To compare the numerical results between the software Ansys^®^, Solidworks^®^ and Inventor^®^, we aimed to discretize the mandible with the same number of elements. Depending on the meshing algorithm of each software package, there was variation in the element numbers. The results of the mandible discretization for the software Ansys^®^, Inventor^®^ and Solidworks^®^ are shown in Table 2.

**Figure 3:**
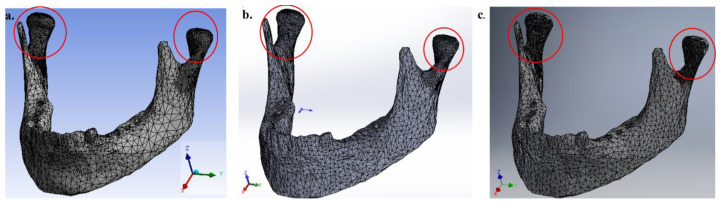
Mesh refinement in the mandible geometry without the molar teeth on the right side: (**a**) Ansys^®^, (**b**) SolidWorks^®^ and (**c**) Inventor^®^ software.

For consistency in the numerical results, it is possible to observe that in the local refinement region, the average size of the element was ~1.15 mm. The final mesh of the whole mandible for each FEM software package can be observed in Figure 3. These values were obtained after performing a convergence criterion for von Mises stress.

It should be noted that, in traditional FEM, 80% of the time is spent on fine-tuning a mesh in order to get a good numerical result. The software SimSolid^®^ (2019, Altair, Troy, MI, USA) provides meshless FEA, but it operates very differently. It was not necessary to generate a mesh, so instead we used high-order functions that are locally adapted to refine the solution to simulate a physical model.

### 2.3. Loads and Boundary Conditions

In total, three variants of the geometry model of the mandible with and without teeth were investigated: Model A—right and left lateral area without molars; Model B—right lateral area without molars; and Model C—the entire mandible without teeth. To simulate chewing, different vertical loads (50, 100, 150 and 150 N) were applied in these areas in the negative direction of the *Z*-axis. To numerically calculate the mechanical stimulus of these chewing forces, zero displacements in all directions were prescribed on the head of the mandible. Figure 3 shows the boundary conditions and the chewing forces to be applied in the FEM software and the meshless software.

## 3. Results

Linear analyses presented a relevant increase in the von Mises stress values regardless of cortical bone and teeth properties. In all simulations, it is also possible to observe that the von Mises stress maximum value was at the condylar neck for all models (A, B and C). It is also possible to observe that FEM simplifications, element size and local refinement of the mesh promoted amplification in the von Mises stress values, and in the area in which the stress dissipates for the FEM software and the meshless software.

**Figure 4:**
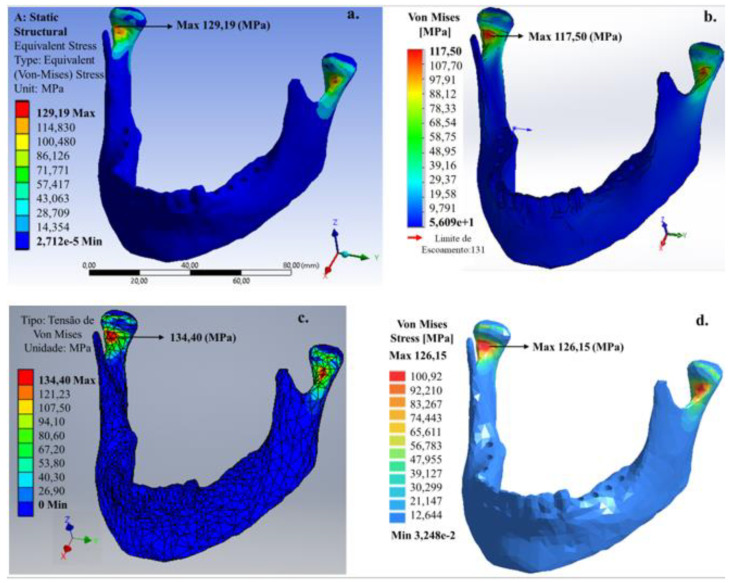
Von Mises stress distribution in the mandible applying 200 N load on the right and left lateral area of the lower dental arch: (**a**) Ansys^®^, (**b**) SolidWorks^®^, (**c**) Inventor^®^ and (**d**) SimSolid^®^ software.

For the 200 N vertical load, applied to the left and right areas without molar teeth (Figure 4), the von Mises stress maximum value that was mainly concentrated at the condylar neck was 126.19 (Ansys^®^), 117.50 (SolidWorks^®^), 134.40 (Inventor^®^) and 126.15 MPa (SimSolid^®^). The value predicted for the maximum von Mises stress by the SimSolid^®^ software (meshless) was consistent with the FEM software.

**Figure 5:**
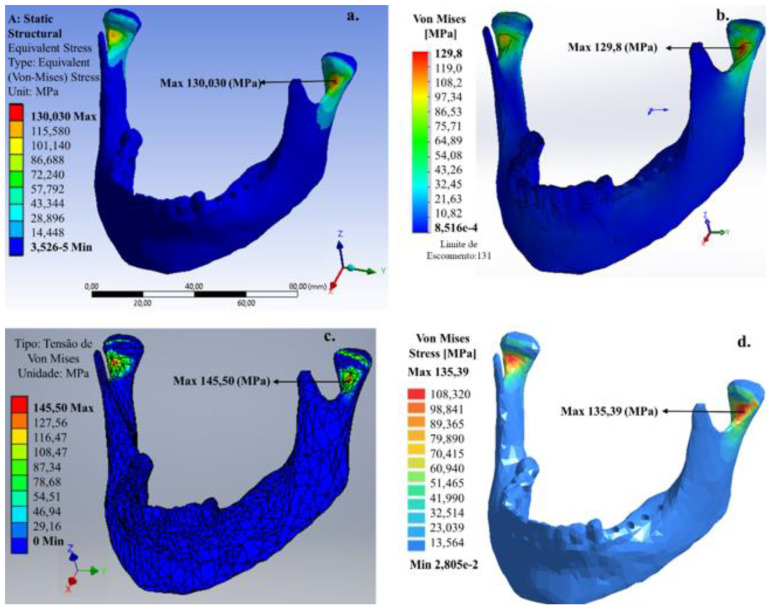
Von Mises stress distribution in the mandible applying 200 N load on the right lateral area of the lower dental arch: (**a**) Ansys^®^, (**b**) SolidWorks^®^, (**c**) Inventor^®^ and (**d**) SimSolid^®^ software.

For all software, the predicted values for the maximum von Mises stress developed for this chewing in the condylar neck were close to the limit of a human bone ~130–190 MPa [17]. The location of the maximum von Mises stress in the internal region of the condylar neck is consistent with the fact that this region is more common to have a fracture of the mandible occur. Figure 5 shows the mandible for a patient missing molar teeth in the right lateral area for the 200 N vertical load.

**Figure 6:**
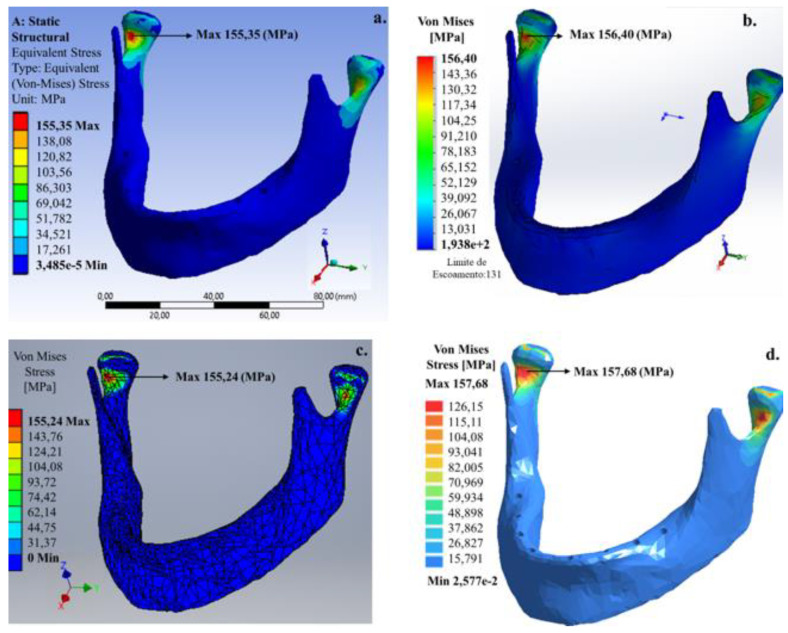
Von Mises stress distribution in the mandible applying 200 N load on the total area of the lower dental arch: (**a**) Ansys^®^, (**b**) SolidWorks^®^, (**c**) Inventor^®^ and (**d**) SimSolid^®^ software.

When comparing with the bilateral chewing, the unilateral chewing in the region without molar teeth caused an increase in the values of maximum von Mises stress. This increase was ~1 (Ansys^®^), ~12 (SolidWorks^®^), ~11 (Invertor^®^) and ~9 MPa (SimSolid^®^). Additionally, the location of the region with the maximum von Mises stress occurred in the condylar neck on the right side. Since the condylar neck is the anatomically weakest region of the mandible, fractures may occur due to stress concentration by indirectly transmitted force. In contrast to bilateral chewing, the predicted value of the maximum von Mises stress by the SimSolid^®^ software (Meshless) was higher than that predicted by the FEM software. Figure 6 shows an edentulous patient in the lower dental arch with application of the 200 N vertical load.

For this model, the maximum von Mises stress occurred similarly to the bilateral model (Model A), but with a better stress extension on the stresses in the condylar neck on the mandible. Von Mises stresses for this model with the total absence of teeth that occurred in both the internal region of the condylar neck were ~155 (Ansys^®^), ~156 (SolidWorks^®^), ~155 (Invertor^®^) and ~157 MPa (SimSolid^®^). Like Model B, SimSolid^®^ software presented the highest values for von Mises stress.

For the 200 N vertical load, the numerical results obtained by the meshless method were consistent with those obtained from the FEM. Thus, the vertical loads 50, 100 and 150 N were applied to all mandible models to confirm the applicability of SimSolid^®^ software. The results were compared with the FEM software. The von Mises stress and relative error values obtained are shown in Table 3.

Usually, the stress deviation can be explained through the geometric nonlinearity and abrupt variation in element size associated with the computational efficiency of each simulation software. An imported mandible with the teeth geometry obtained by a computed tomography scan may present errors in the generation of the 3D model, which need to be repaired or reduced, otherwise, there will be the generation of an inadequate geometry or an extremely complex numerical simulation. On the other hand, when there are abrupt variations in the geometry or shape and element size, a solver error can be generated, thus generating inaccurate numerical results. For these reasons, it is possible to affirm that SimSolid^®^ software is the most viable option since there is the possibility of considering the mechanical properties of each piece of the total model and requiring less time for the pre-processor and processor of the problem resolution.

Three conventional software simulations where mesh generation is necessary (Ansys^®^, SolidWorks^®^ and Inventor^®^) and a meshless software (SimSolid^®^) were compared. The comparison showed similar behaviors in the highest concentration of tension regions, namely the upper edge of the mandibular condyle, which is the temporomandibular joint position.

Regarding the tension values, the percentage error between each software package was less than 10%. Concerning the meshless algorithm (SimSolid^®^ software), it is possible to observe that the highest stress concentration obtained has a maximum percentage error of less than 7% when compared to other FEM software.

## 4. Discussion

In this study, computer-based technologies were used to obtain the place and the stress value of the mandibular structure. Thus, the highest stress concentrations were generated by masticatory forces during the use of dental prosthesis rehabilitation in edentulous patients [22].

For this reason, an integrated environment to improve the process to determine the best dental prosthesis fixation points and their masticatory force influences and, consequently, to prevent pain and discomfort was developed.

An approach integrating a facial computed tomography (CT) scan in a CAD/CAM image treatment workflow for complete-mouth implant-supported rehabilitation for patients with a total or partial lack of the mandibular structure was shown. It can be noted that the use of CT images can create a virtual 3D model which provides a powerful tool to determine the most adequate place to fix the dental retention pin such as intramucosal inserts (Muchor^®^), whether by virtual reality or a physical model produced by rapid prototyping.

The complete or partial discontinuity of the mandibular bone causes direct or indirect forces variations during the mastication. Further, the patterns of strain and stress are strongly affected by the material properties of the studied structure, as the bone materials are highly anisotropic, where the crystals of hydroxyapatite (phosphate mineral) with a high Young’s modulus form within the collagen and elastic protein fiber with a low Young’s modulus. Considering the information given, it can be concluded that any attempt to obtain a numerical model will be an extremely laborious task.

## 5. Conclusions

The results showed the maximum stress value of 156.40 MPa for a 200 N load distributed in the total area of the dental arch in the SolidWorks^®^ software, due to both the jaw geometry and load value, and also its place associated with discretization solver and the total number of elements used in the SolidWorks^®^ software.

The minimum stress value of 29.57 MPa for a 50 N load was distributed in the right and left sides of the dental arch area in the SolidWorks^®^ software. It can be attributed to a smoother refining process used by this algorithm.

Finally, it is possible to conclude that the use of SimSolid^®^ software proved to be better when identifying the higher tension generated by the dental prosthesis in the facial skeleton, and considering its geometric complexity, the computational efficiency must be highlighted.

## Figures and Tables

**Table 1:** Mechanical properties assigned to the analyzed materials [18,19].

Material	Young’s Modulus	Poisson’s Ratio	Density (g/cm^3^)
	(MPa)		
Cortical bone	14,000	0.33	1.85
Teeth	72,700	0.33	2.97

**Table 2:** Results of the size and number of elements of the mesh generated in the Ansys^®^, SolidWorks^®^ and Inventor^®^ simulation software.

Software	Ansys^®^	SolidWorks^®^	Inventor^®^
Maximum element size (mm)	3.26	3.58	3.84
Minimum element size (mm)	1.05	1.19	1.23
Total nodes	199,713	162,936	115,540
Total elements	126,590	106,545	100,974

**Table 3:** Results of the von Mises stress and the percentage error between the software Ansys^®^, SolidWorks^®^ and Inventor^®^ compared to the software SimSolid^®^.

Von Mises Stress Results								
Model	Vertical Force (N)	Stress (MPa)				Relative Error (%)		
		SimSolid^®^	Ansys^®^	SolidWorks^®^	Inventor ^®^	Ansys^®^	SolidWorks^®^	Inventor^®^
Right and left lateral area	50	31.53	32.29	29.57	33.68	2	6	7
	100	63.07	64.59	59.01	67.21	2	6	7
	150	94.61	96.89	88.32	100.86	2	7	7
	200	126.15	129.19	117.51	134.42	2	7	7
Right lateral area	50	33.91	32.51	32.45	33.58	4	4	1
	100	67.73	65.01	64.89	70.96	4	4	5
	150	101.54	97.52	97.34	108.25	4	4	7
	200	135.34	130.03	129.82	145.52	4	4	7
Total area of the lower dental arch	50	39.42	38.83	39.09	38.86	1	1	1
	100	78.83	77.67	78.18	77.67	1	1	1
	150	118.26	116.51	117.30	116.42	1	1	2
	200	157.68	155.35	156.40	155.24	1	1	2
